# The Yellow Oxide of Mercury

**Published:** 1882-10

**Authors:** W. W. Seely

**Affiliations:** Professor of Ophthalmology and Otology in the Medical College of Ohio


### ARTICLE II.

**THE YELLOW OXIDE OF MERCURY.** By W. W. SEELY, M. A., M. D., Professor of Ophthalmology and Otology in the Medical College of Ohio.

For quite a number of years all oculists and a large proportion of the general profession have been aware of my advocacy of the abandonment of the ordinary astringent-caustic treatment of all conjunctival troubles. I am quite correctly represented (so far as it goes) by the author of an article in a recent number of your journal* on "The Yellow Oxide of Mercury in Ophthalmic Practice," as substituting for astringents and caustics the yellow oxide and vaseline. And I have repeatedly stated that my satisfaction in treating conjunctival affections dates from this period. Except in a journal devoted to general medicines it would be quite superfluous to state that the introduction of the impalpable powder, the yellow oxide of mercury belongs to Pagenstecher; the ointment made with it, however, was usually very strong, 20 to 60 grains to the ounce (of lard) and its use almost restricted to the late stages of corneal affections. Its use in this country had (till my advocacy) been very limited. My devise of vaseline as a vehicle for it along with the recommendation of weaker preparations (8 to 10 grains to the ounce) as well as its use in the first stages of corneal affections, and all forms of conjunctival inflammations, have quite put a new life into it. All I claim (and I am supported in the claim by the priority of my articles) is the use of vaseline as a vehicle, weaker preparations, its use in corneal troubles from their inception, not waiting for a subsidence of the inflammation; its adaptation to conjunctival affections.

---

* Hotz, May, 1882, vol. xliv, No. 5.

Some two years or more, after I had made known this "adaptation," Mr. Baden, Guy's hospital, London, discovered the virtues of the yellow oxide and vaseline in conjunctival troubles, thinking it was new and original, though a glance at the current literature would have shown that it had already been advocated and reported upon by myself.

(See reply of the editor of *Centralblatt für Prakt, Augenheilk'd* to Dr. (now Prof.) Fuchs, in regard to vaseline as a vehicle, etc.)

Each year's experience had added support to my views first enunciated on the non-astringent caustic treatment of conjunctival affections.

The author (Hotz) of the article above alluded to, seems to think my "cure-all" claim extreme, but even if it were a proper statement of my position there is nothing very peculiar in it. A reference to Dr. Wilson's (Bridgeport, Conn.) report will show the position taken in routine practice by the adherents to the old astringent-caustic plan. This one uses nitrate of silver, that one sulphate of copper, another tannin, etc., or changes are rung on two or three astringents. I reply that I never use astringents under any circumstances, my "routine" being yellow oxide of mercury and vaseline. Wherein does my practice differ from that of others? Undoubtedly only in this, that my conjunctival remedy found its first field in, and is regarded as belonging to, corneal affections. I simply substitute the yellow salve for astringents, claiming that astringents may be, and should be, abandoned *in toto*.

I am quite aware of the revolutionary character of my position, but it is receiving support continually, and will be established. There is no man bold enough to claim that the astringent-caustic plan is a success. Any plan equally successful, but freed from the disadvantages of the old one is a gain. So far no one has claimed that the yellow oxide is not equal in efficiency to astringents and caustics. That it is free from these drawbacks needs no argument, when I state that, properly prepared, the salve causes no pain or inconvenience. But my non-astringent caustic plan embraces more than the use of the yellow oxide. I do not regard the yellow oxide in the light of "a powerful local stimulant and alterative, promoting the absorption of the products of inflammation and encouraging the process of repair." (Hotz.) Certainly not in the language quoted.

Now, whoever advocated this remedy under the above language may be, and I am not quite pursuaded he is not, claiming something for it that it does not possess, at least not beyond many other substances. I have no doubt the author of the language quoted does not differ from many others, when he uses it as a "stimulant and alterative." Further, I have no doubt that herein lies his unwillingness to "follow the example of a recent writer."

I am not at all pursuaded the yellow oxide surpasses other substances as an irritant and absorbant. It may possess their attributes, mysterious as may be the use of them, but when I put it on a violently inflammed conjunctiva I certainly do not have in view the production of irritation and absorption. Omitting any special theories of inflammation, an apparently important desideratum is the contraction of blood vessels. I do not think I am wrong when I state that the yellow oxide does bring about contraction of the blood vessels in the pathological state in question. I have seen the sclera become so pale and such dryness produced that I have discontinued its use for the time.

We all know that the astringents contract blood-vessels, but along with the knowledge of that fact goes the knowledge of the other, that the primary contraction is likely to be minimum, the reaction (or dilatation) maximum. Who has learned the secret of reversing the above reaction ? We have all been brought up in the same latitudinarian school, teaching "salutary reaction," "increased circulation," "carrying off of the morbid products, etc., etc.

The absolute necessity for nitrate of silver in purulent states of the conjunctiva has been almost as fixed as existence itself, and I thoroughly recognize the shock produced by a flat denial supported incontrovertably by clinical experience, and now the latter backed up by mycology.

In my therapeutic investigations the purulent state was the last in which I abandoned the astringent-caustic treatment, while it should have been the first.

Sometime after my hardihood in asserting that purulency did not demand astringents and caustics, Professor Becker, of Heidelberg, went a step further and claimed that only cleanliness was needed. It can then, I believe, be considered demonstrated that purulency does not require astringents and caustics. If I am not mistaken we have been in the habit of regarding the purulent state as really but a grade higher in the inflammatory list than a catarrhal. Taking then but the logic of all the books, and only that logic, and where does it lead us? It is so clear "the wayfaring man though a fool need not err therein." Trachomatous conjunctivitis consists in the formation of new growths in and at the expense of the pre-existing conjunctival elements, a process accompanied by more or less catarrhal conjunctivitis, and it is now rendered quite probable that even the trachomatous bodies are due to a parasite (the trachoma coccus of Prof. Huburt Sattler, of Erlangen). It follows if the chief of conjunctival inflammations (laying aside now any causes) does not require astringents, caustics, why should the minor forms? My clinical studies antedated the birth of medical mycology proper. In brief, I accidentally discovered in treating the corneal complications of trachoma, that the lid trouble likewise subsided in a remarkable manner under the yellow oxide, from which I was finally led to abandon the usual remedies. By inference I passed to other forms with of course more striking results, since this element of self-limitation is more marked. I may then state my position as follows: Absolute clinical conviction, subsequently my theory resting on deductions from mycology, and lastly the scientific basis as laid by investigations from various sources and recognized, I fancy, by all. I venture to claim that no part of either general or ocular therapeutics rests on a more solid foundation.

The old salves and collyria of the bi-chloride of mercury were ahead of their age, for it is only modern science that has taught us why they are efficient, by showing that it only requires one part of the bi-chloride to 20,000 of water to kill the vegetable growths of ordinary decomposition. My rationale of the action of the yellow oxide has been that in the presence of the sodium chloride furnished by the tears, a certain amount of bi-choride is formed. Whether this is correct or not the experiments I am now making will determine, as they will also I think, the influence of the yellow oxide on the parasites of the various conjunctival secretions. I have assumed that in the remedy advocated I had a powerful parasiticide, as I know I had a contractor of blood vessels, which two actions I have claimed as proper for a remedy in conjuntival inflammations.

As I have so frequently stated in other places, I believe Professor Leber gave the only rational explanation of the action of astringents when he said it is parasiticidal. Now all investigations go to show that these materials stand quite at the bottom of the list in checking the growths of ordinary decomposition. (See table in Real Encyclopœdie.)

To carry out my ideas in full necessitates one more revolution. For nearly two decades atropine has been the adjuvant of the astringent-caustic treatment, especially in the more important forms of conjunctival inflammation, such as the trachomatous and the purulent. All investigations prove that the effect of atropia is dilatation of blood vessels from paralysis of the muscle wall. Now if inflammation is really little more than exaggerated nutrition, the blood-vessels are enlarged, even new ones are formed, and the problem is to contract and obliterate them, which cannot be done by a paralysis of thin walls. The same thing holds true in non-vascular structures, since the hypernutrition is here represented by both an increased lymph supply, and the blood (in some cases) of new formed vessels. It is claimed by very high authority that the lymph vessels are directly connected with the blood-vessels, at any rate their circulation stands in close relation to that of the blood.

In the case of the cornea, every one knows the first phenomenon of an inflammation of this structure is an accumulation of leucocytes; further on a consequent crowding together, strangulation, even necrosis of the tissue. The problem here too is precisely the same, to check the supply. This of course is not the only problem, but the first. Now corneal complications represent the sole "mode of death" in conjunctival inflammations. I may be pardoned then for my conclusion, other things being equal, for resorting to the same general principle in the management of inflammation of these two structures.

For a series of years I have resorted more and more exclusively to the use of the myotics which contract blood-vessels, such as eserin, in corneal affections, instead of mydriatics, such as atropine, and with constantly increasing satisfaction.

Now, if I carry out the principles I am compelled, if I use one or the other, to resort to a myotic (such as eserin) in conjunctival affections. From the present stand-point then of our knowledge I feel justified in insisting upon the principles I have laid down, besides my clinical experience so far has added, for me, the demonstration of this truth. As will have been seen, my chief object has been to enunciate certain foundation principles, using given remedies as my text. I am far in reality from believing that anything like perfection has been reached, but I do believe that the steps taken, while out of the beaten path, will help form a new one far plainer and better.

